# Fournier’s Gangrene With Retroperitoneal Extension as the First Manifestation of the Human Immunodeficiency Virus (HIV)/Acquired Immunodeficiency Syndrome (AIDS)

**DOI:** 10.7759/cureus.20517

**Published:** 2021-12-19

**Authors:** Andrés Felipe Herrera Ortiz, Juan G Arámbula, Valeria Del Castillo, Yasmin Eltawil, Bassel Almarie

**Affiliations:** 1 Radiology, Universidad El Bosque, Bogotá, COL; 2 General Medicine, Universidad El Bosque, Bogotá, COL; 3 Head and Neck Surgery, University of California San Francisco, San Francisco, USA; 4 General Surgery, Kantonsspital St. Gallen, Saint Gallen, CHE

**Keywords:** fournier’s gangrene, necrotizing fasciitis, severe sepsis, sigmoid-end colostomy, radical orchiectomy, retroperitoneal extension, retroperitoneum, aids, hiv

## Abstract

Fournier’s gangrene (FG) is an atypical, life-threatening polymicrobial infection characterized by the rapid destruction of soft tissue, predominantly in the perineal region. Retroperitoneal spread of FG represents an uncommon condition described in a few case reports, and its presentation as the first manifestation of the human immunodeficiency virus (HIV)/acquired immunodeficiency syndrome (AIDS) is even more infrequent. Here, we present the case of a 40-year-old male who was admitted to the emergency department with a low-grade fever of 37.8°C, abdominal pain, and four-day history of sharp, bilateral testicular pain and swelling. On physical examination, the patient was hypotensive with necrotic tissue in the perineum. A computed tomography study displayed an extensive retroperitoneal spread of suspected FG. Due to the massive spread of the infection, an HIV test was requested, yielding positive results, which indicated that HIV/AIDS had first manifested as FG with retroperitoneal extension. This is an extremely rare initial presentation of HIV/AIDS. To treat the patient and address the severe necrosis, a peritoneal lavage, surgical debridement, right orchiectomy, and colostomy were performed. After the procedure, antiretroviral therapy was established with tenofovir, emtricitabine, and efavirenz.

## Introduction

Fournier’s gangrene (FG) is an atypical, life-threatening polymicrobial infection caused mainly by *Escherichia coli*, *Klebsiella*, *Staphylococcus aureus*, *Streptococcus*, and anaerobes. The infection is characterized by devastating, rapid soft tissue destruction predominantly affecting the perineal, perianal, and genital regions [1]. FG represents less than 0.02% of all hospital admissions in the United States, affecting mainly males with an incidence rate of 1.6 per 100,000 males. The condition typically presents with a mortality rate ranging between 20% and 50% in most cases [2-4]. While FG is well-documented in the literature, the retroperitoneal spread of the disease represents an uncommon condition described in only a few cases. In this report, we present an even more infrequent description of FG as the first manifestation of the human immunodeficiency virus (HIV)/ acquired immunodeficiency syndrome (AIDS) and its successful management.

## Case presentation

A 40-year-old male construction worker with no previous medical history presented to the emergency department with a 12-hour history of fever and abdominal pain and a four-day history of sharp, bilateral testicular pain and swelling.

**Figure 1:**
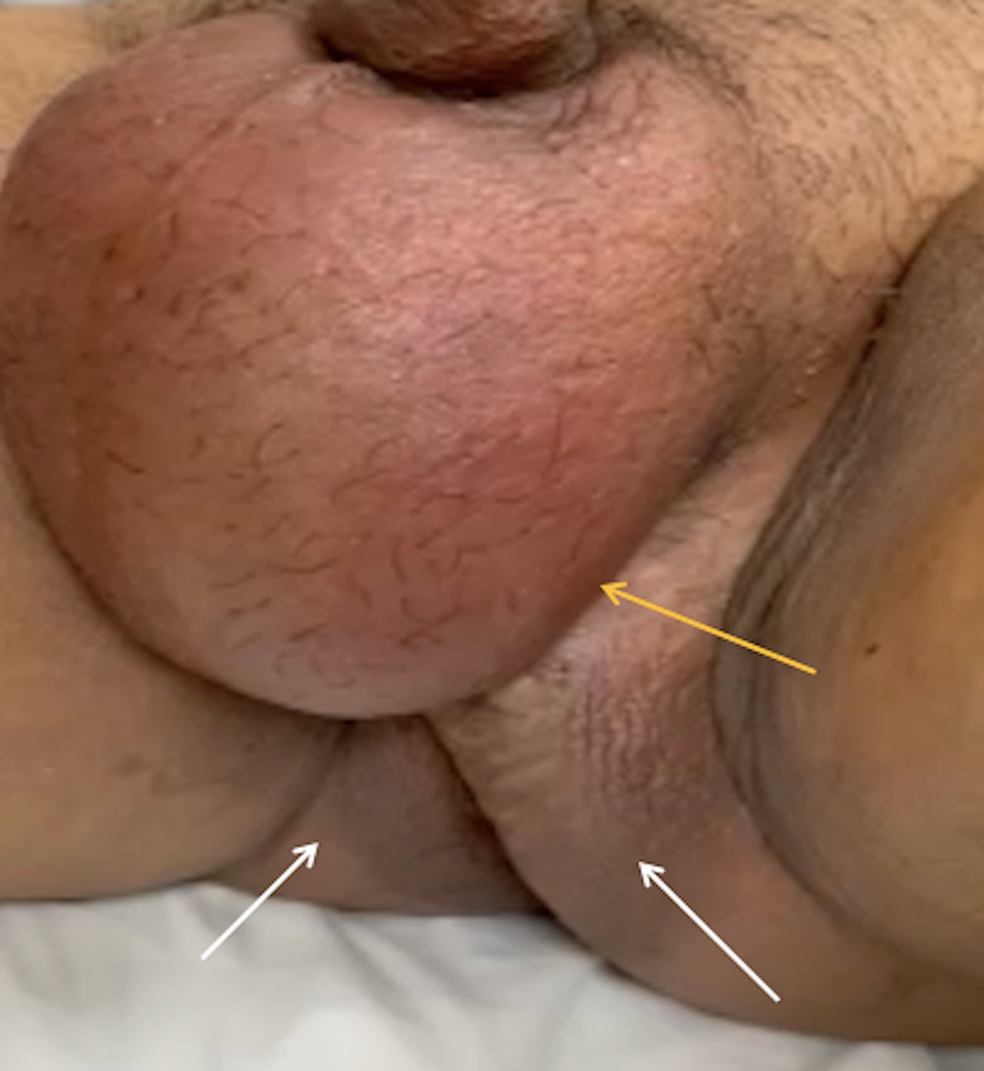
Perineal region on admission. The image displays an enlarged, swollen, edematous scrotum (orange arrow) and patches of gangrene with necrosis on both sides of the perineum (white arrows).

On assessment, the patient’s temperature was 37.8°C, blood pressure 85/46 mmHg, pulse 120 beats per minute, and respiratory rate 24 breaths per minute with no shortness of breath. Upon physical examination, the patient appeared to be distressed but was fully aware of his spatial and temporal surroundings. An abdominal examination revealed a distended abdomen with guarding, rebound, and severe tenderness in the hypogastrium. The patient had normal bowel sounds. Genital examination revealed bilateral necrotic patches on the perineum, associated with a foul odor, crepitus, and an enlarged, edematous scrotum (Figure 1). The remainder of the physical examination showed unremarkable findings.

After the initial examination, laboratory tests were ordered, showing anemia, leukocytosis with a neutrophilic predominance, and elevated creatinine levels (Table 1). Based on the findings, the Sequential Organ Failure Assessment score was calculated as 4 points, which indicated that the patient was in sepsis.

**Table 1:** Laboratory values on admission. CRP: C-reactive protein; PaO_2_/FiO_2_: pressure of arterial oxygen to fractional inspired oxygen concentration; BUN: blood urea nitrogen

Variable	On admission	Reference range
Hemoglobin (g/dL)	11	13.8–17.2
Hematocrit (%)	35.3	41–50
Differential count (%)		
Neutrophils	92	39.3–73.7
Lymphocytes	4.9	18–48.3
Monocytes	2.2	4.4–12.7
Eosinophils	0.5	0.6–7.3
White blood count (µL)	21,230	4,500–11,000
Platelet count (per mm^3^)	166,000	150,000–450,000
CRP (mg/L)	29.23	<1
Potassium (mEq/L)	3.41	3.5–5
Carbon dioxide (mEq/L)	33.5	22–29
PaO_2_/FiO_2_ ratio	369	>300
Creatinine (mg/dL)	2.12	0.7–1.3
BUN (mg/dL)	60.2	6–24

**Figure 2:**
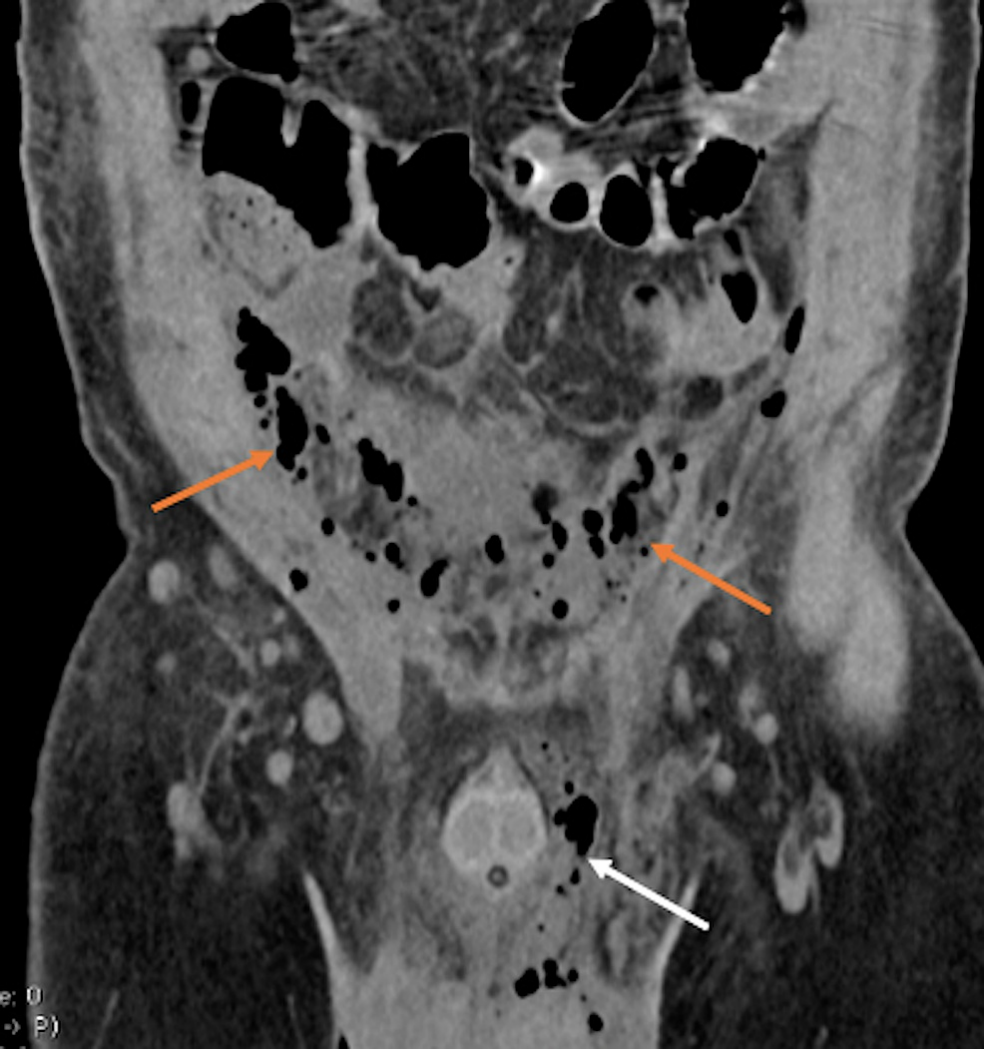
Non-contrast abdominal CT in coronal view. Multiple emphysema foci spreading from the genital area (white arrow) to the peritoneum (orange arrows). These findings are consistent with an intraperitoneal extension of FG. CT: computed tomography; FG: Fournier’s gangrene

**Figure 3:**
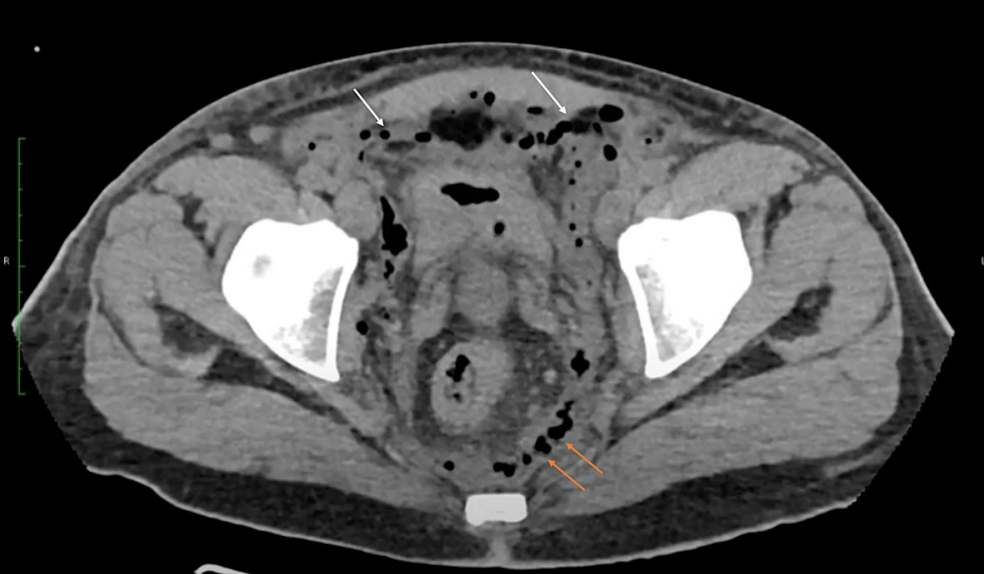
Non-contrast abdominal CT in axial view. Intraperitoneal emphysema (white arrows) with retroperitoneal extension (orange arrows). These findings are consistent with the intraperitoneal and retroperitoneal extension of FG. CT: computed tomography; FG: Fournier’s gangrene

A non-contrast computed tomography (CT) was performed to assess the necrotic tissue extension. The imaging revealed multiple foci of emphysema and inflammation affecting the genital area and extending to the peritoneum and retroperitoneum (Figures 2, 3).

Based on the perineal necrotic patches detected on physical examination, we established the diagnosis of FG. The presence of hypotension with organ dysfunction, acute abdomen, and perineal and retroperitoneal necrotic foci raised the suspicion of septic shock resulting from retroperitoneal FG. An HIV test was requested to detect the underlying causes of the extensive necrotic compromise, leading to the diagnosis of HIV infection with a viral load of 460,630 copies/mL and CD4 of 87/mm^3^. The patient later revealed that he had been practicing unprotected sexual activity and may have contracted the virus from one of his sexual partners.

**Figure 4:**
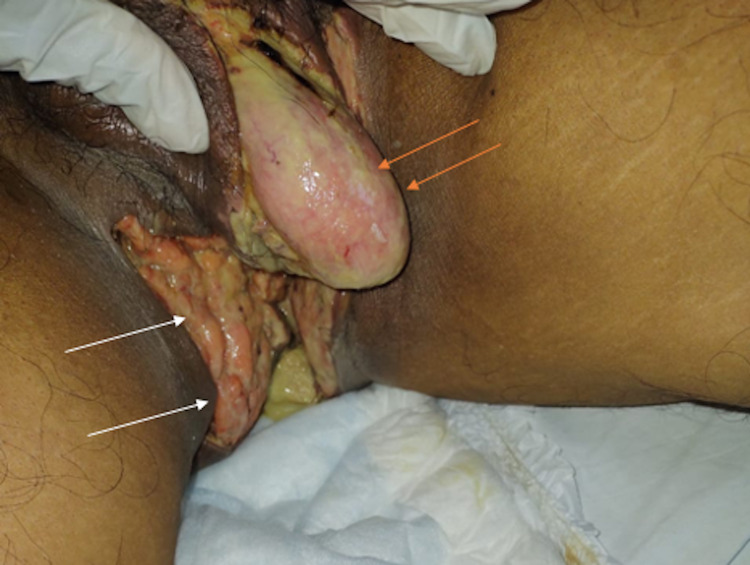
Perineal area after debridement. A right orchiectomy was performed and all the devitalized tissue was removed in the perineum (white arrows) and scrotum (orange arrows).

The patient’s acute presentation and CT findings indicated an emergency explorative laparotomy. This procedure revealed that the FG was severe and had spread to the peritoneal and retroperitoneal space. Therefore, peritoneal lavage was performed to facilitate the removal of the infected, necrotic tissue, followed by surgical debridement. The necrotic tissue in the perineal area, left ischioanal fossa, and left gluteus was carefully removed in a stepwise fashion until there was bleeding from small vessels, indicative of vital and perfused tissue (Figure 4). While the penis and the left testicle were intact, the gangrene had compromised the right testicle, spermatic cord, and reached the anal sphincter. Therefore, a right radical orchiectomy was performed to address the compromised testicle, and a temporary colostomy was constructed due to the anal sphincter involvement. Purulent fluid was sampled intraoperatively for culture showing extended-spectrum beta-lactamases *E. coli*.

**Figure 5:**
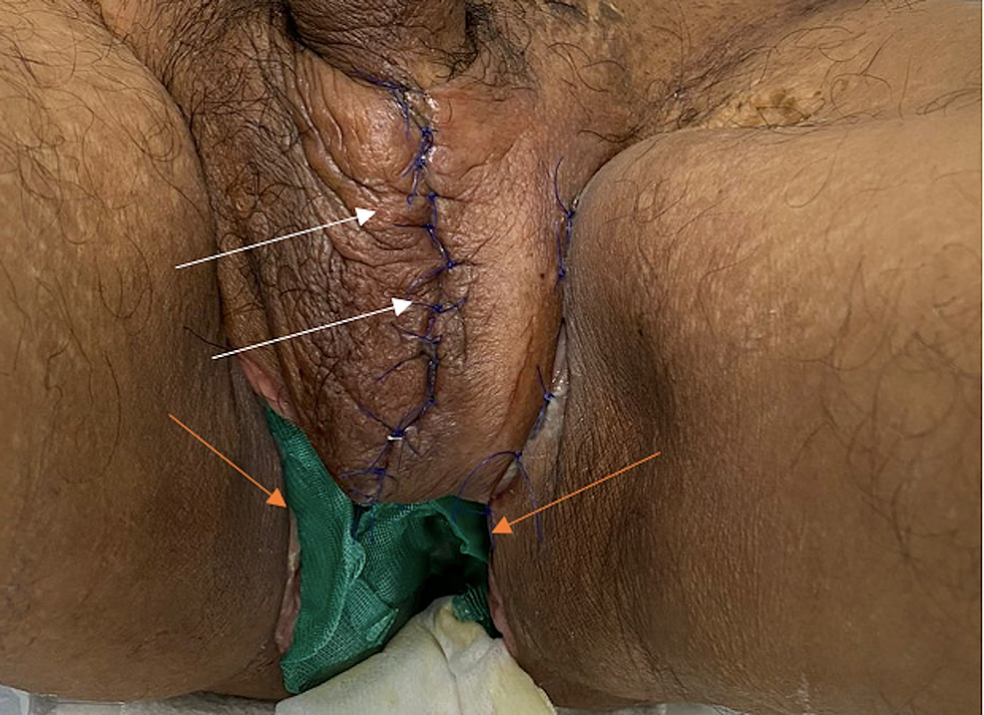
Post debridement evolution. Primary intention closure in the scrotum with an adequate healing process (white arrows) and secondary intention closure in the perineum area (orange arrows).

After the procedure, the patient was transferred to the intensive care unit under antiretroviral therapy (tenofovir, emtricitabine, efavirenz) and vasopressor support. The exposed areas were covered with saline-soaked gauze, and the infection was treated with empiric antimicrobial therapy consisting of meropenem and clindamycin. On postoperative day five, the patient was stabilized and transferred from the intensive care unit to the surgical floor. The hospital’s plastic surgery team evaluated the patient’s condition and performed a primary intention closure in the scrotum and a secondary intention closure of the debrided area in the perineum on postoperative day 15 (Figure 5). On postoperative day 23, the patient showed satisfactory development and was discharged.

Postsurgically, a multidisciplinary ambulatory action plan was established with plastic and general surgery. Additional control appointments with the infectious disease department, psychology, and social services were set up to address the recent HIV/AIDS diagnosis. After two months of follow-up, the patient returned to his baseline clinical status without retrieving anal tone; consequently, the colostomy was continued. Erectile function and ejaculation were preserved, and the cosmetic outcome was satisfactory.

## Discussion

Retroperitoneal involvement is an uncommon manifestation of FG, seen in less than 30% of all cases. Furthermore, its clinical connection to HIV is even more infrequent [5]. Retroperitoneal FG has a higher incidence rate among women, as suggested by Czymek et al., due to the internal localization of the female genitalia compared to the male genitalia, which leads to easier retroperitoneal spread [5]. Conversely, the clinical picture of HIV infection is varied and usually manifests at admission as acute retroviral syndrome presenting with flu-like symptoms [6]. In this case, we report the presence of FG with retroperitoneal involvement serving as the first manifestation of HIV/AIDS [5].

There are specific risk factors that predispose to FG. These include low socioeconomic status, poor personal hygiene, alcohol use disorders, the use of corticosteroids, diabetes, immunodeficiencies such as HIV/AIDS, and cancer [7]. In this report, HIV/AIDS was associated with the retroperitoneal extension of FG, which is a relationship that has been described in close to 11 cases in Western literature [8]. In 2014, Ngugi et al. reported that HIV infection was the most common condition associated with FG in Africa (16.4%), followed by diabetes mellitus and alcoholism (11%) [9]. However, Ayumba et al. reported that only 4% of FG infections were associated with HIV as a comorbidity [10]. Despite the previous association of FG with HIV/AIDS, only one case report published in 2007 by Chazan et al. described FG with retroperitoneal extension as the first manifestation of HIV/AIDS [11].

It is essential to highlight that HIV/AIDS is not the cause of FG. However, we hypothesize that HIV may be a risk factor for extensive dissemination of the infection. This is due to an impaired immune system that is unable to regulate the dissemination of the disease, which often leads to a more severe clinical picture, as shown in this report.

FG is diagnosed in the same way in patients with and without HIV. The diagnosis is based on physical examination where necrotic tissue is identified in the perineal, perianal, or genital regions. Imaging methods such as conventional radiography, point-of-care ultrasound, CT, and magnetic resonance imaging (MRI) can often detect the presence of soft tissue edema, soft tissue emphysema, and collections that help confirm the diagnosis of FG [2,4]. CT or MRI are frequently used as guides for appropriate surgical planning because they provide information regarding the extent of the necrosis.

The presence of HIV does not change the treatment pathway of FG. Treatment includes broad-spectrum antibiotics, emergent surgical debridement of all necrotic tissue, appropriate hemodynamic resuscitation with intravenous fluids, and vasoactive medications when required [2,4]. It is essential to highlight that to ensure that all necrosis has been removed, the debridement of the necrotic tissue must be done until bleeding from small blood vessels is observed [12,13]. In some scenarios, orchiectomy, colostomy, or cystostomy may be required; nevertheless, there is no clear indication in the literature on when to perform these procedures [13]. After surgical debridement, it is highly recommended to cover the exposed area with saline-soaked gauze and change it frequently during the day [13].

Currently, the literature shows the same rate of FG recovery in patients with and without HIV [8,14]. However, few studies suggest an increased mortality rate due to FG for every three-fold rise in HIV viral load, while others indicate that the risk of death depends on the patient’s status within the emergency room rather than the findings of laboratory tests [8,15,16].

## Conclusions

In conclusion, FG with severe extension must raise the suspicion of an underlying condition such as HIV/AIDS. The diagnosis of FG in the context of HIV is the same as in the general population; therefore, imaging modalities such as abdominal and pelvic CT scans remain essential to identify the spread of necrosis. To achieve successful treatment of FG, all the necrotic tissue must be removed until there is bleeding from small vessels, indicative of vital and perfused tissue.
